# City-Wide Traffic Flow Forecasting Using a Deep Convolutional Neural Network

**DOI:** 10.3390/s20020421

**Published:** 2020-01-11

**Authors:** Shangyu Sun, Huayi Wu, Longgang Xiang

**Affiliations:** State Key Laboratory of Information Engineering in Surveying, Mapping and Remote Sensing, Wuhan University, Wuhan 430079, China; shangyu_sun@whu.edu.cn (S.S.); wuhuayi@whu.edu.cn (H.W.)

**Keywords:** city-wide traffic flow forecasting, multi-branch prediction network, deep learning, external factors fusion, taxicabs GPS trajectories

## Abstract

City-wide traffic flow forecasting is a significant function of the Intelligent Transport System (ITS), which plays an important role in city traffic management and public travel safety. However, this remains a very challenging task that is affected by many complex factors, such as road network distribution and external factors (e.g., weather, accidents, and holidays). In this paper, we propose a deep-learning-based multi-branch model called TFFNet (Traffic Flow Forecasting Network) to forecast the short-term traffic status (flow) throughout a city. The model uses spatiotemporal traffic flow matrices and external factors as its input and then infers and outputs the future short-term traffic status (flow) of the whole road network. For modelling the spatial correlations of the traffic flows between current and adjacent road segments, we employ a multi-layer fully convolutional framework to perform cross-correlation calculation and extract the hierarchical spatial dependencies from local to global scales. Also, we extract the temporal closeness and periodicity of traffic flow from historical observations by constructing a high-dimensional tensor comprised of traffic flow matrices from three fragments of the time axis: recent time, near history, and distant history. External factors are also considered and trained with a fully connected neural network and then fused with the output of the main component of TFFNet. The multi-branch model is automatically trained to fit complex patterns hidden in the traffic flow matrices until reaching pre-defined convergent criteria via the back-propagation method. By constructing a rational model input and network architecture, TFFNet can capture spatial and temporal dependencies simultaneously from traffic flow matrices during model training and outperforms other typical traffic flow forecasting methods in the experimental dataset.

## 1. Introduction

City-wide traffic flow forecasting is a significant function of the Intelligent Transport System (ITS), which plays an important role in city traffic management and public travel safety. In addition, this type of forecasting can help people make better daily travel plans and optimize the allocation of public transport resources. Several mainstream online map websites (e.g., Google Maps, Baidu Maps, Tencent Maps) provide real-time traffic status and traffic status forecasting functions, which have contributed greatly to easing traffic pressure, by receiving a large amount of sensor data.

Predicting the future state of the traffic system is always a challenging task, especially for city-wide traffic flow forecasting, which is related to many complex factors both inside and outside the complex system. City-wide forecasting involves estimation of the overall traffic status of the urban road network compared with single road segment forecasting. Prediction accuracy is prone to be affected by road network distribution but also by weather, accidents, holidays, etc. The urban road network consists of numerous segments and usually covers the entire urban and suburban area. The traffic flow of a specific road segment is simultaneously influenced by the upstream and downstream road segments, as well as adjacent road segments. Thus, city-wide forecasting cannot ignore the complex topological relationships of this urban road network. Furthermore, city-wide forecasting is a typical time series prediction problem, and the long-term temporal dependency of historical observations is hard to capture with traditional forecasting approaches, especially when modelling periods and trends. Moreover, external factors like torrential rain, traffic accidents, important festivals, etc., impact urban traffic conditions. Unfortunately, traditional prediction models cannot solve the above challenges. These models usually treat traffic flows as sequential data and forecast future states based on specific hypotheses but neglect the fact that a traffic system is a non-linear system. Furthermore, they tend to suffer from some limitations, such as their ineptitude in processing outliers, noisy data, or missing values, and their incompetence in handling the problem of dimensionality [1]. Thus, how to improve prediction accuracy is worthy of further discussion.

Statistical methods and neural networks are two typical approaches for traffic flow forecasting. Both of these methods have dominated mainstream research methods in recent years.

Statistical methods are widely used in traffic flow forecasting research. According to the periodicity of traffic state evolutions, some nonparametric models, such as k-nearest neighbours (KNNs), have been utilized to predict traffic speeds and volumes [2,3,4]. Support vector machines (SVMs) [5] and their various extensions [6,7,8] have promoted the improvement of prediction accuracy by capturing the complexity within traffic systems. Some researchers have tried to combine multiple models to enhance model performance and improve traffic flow prediction accuracy for a single road segment [9]. Recognizing the correlations in the successive observations of traffic state evolutions, some researchers brought time-series prediction models into the traffic prediction problem. Autoregressive integrated move average (ARIMA) is a typical method and incorporates several essential characteristics of traffic flow, such as internal correlation (via a moving average) and its effect on short-term future (via autoregression) [10,11,12,13]. Although statistical methods have been widely adopted, they cannot effectively extract inherent spatial and temporal dependencies and remain limited to a single road segment scenario. To predict city-wide traffic flow volume, a large number of independent models must be constructed. This limits the further application of statistical methods in the city-wide traffic flow prediction problem.

Neural networks (NNs) are also employed in the traffic flow prediction problem, due to their strong non-linear fitting ability [1]. For example, the study in [14] combined an ANN with Bayes’ theorem to predict the short-term traffic flow volume on a freeway. The study in [15] designed a statistical and ANN mixed model to forecast traffic flow volume in an urban area. However, ANNs have not been widely used because of their poor generalization ability caused by their inherently shallow architecture. At present, several advanced and powerful deep learning models have been introduced into the academic community to solve traffic flow prediction problems. For example, the work in [16] utilized deep learning methods to predict the traffic status of a local region. The researchers in [17] were the first to adopt Deep Belief Networks (DBNs) into the traffic forecasting field and achieved favourable results. The study in [18] introduced an RBM-RNN model that combined deep restricted Boltzmann machines (RBMs) with a recurrent neural network (RNN), which inherited the advantages of the above two models. The work in [19] constructed a deep-learning-based prediction model and utilized a stack auto-encoder (SAE) to extract local spatiotemporal features. Deep learning models have achieved better results than traditional methods because they possess much deeper and more complicated structures. However, few studies have involved city-wide traffic prediction problems and especially lack enough consideration of both spatiotemporal dependencies and the complex external influences hidden in the traffic system.

This paper proposes a deep-learning-based approach that is applicable to the city-wide traffic prediction problem. This method simultaneously considers the spatial correlation and temporal dependencies of traffic flows in a road network and takes external factors into account. Our contributions can be summarized as follows:

We propose a deep-learning-based city-wide traffic flow forecasting model. This model feeds on the traffic status of the urban road network and can simultaneously extract spatial and temporal features during model training. With the help of multi-branch fusion, this model effectively integrates external factors with the model’s main component, which, to some extent, improves prediction accuracy.

We introduce a novel data pre-processing method, which calculates the traffic flow volume of the entire urban and suburban area based on taxicab GPS trajectories. This method converts the research area into a spatial lattice and produces traffic flow matrices that represent the traffic status of an urban road network. This smaller granularity can precisely reflect a realistic traffic flow volume compared to a single road segment.

We propose an encoding method for external factors, which encodes weather, accidents, and holidays into one-hot encoded alike vectors. The input vectors are embedded into a hidden layer through a two-layer fully connected neural network and expanded to a high-dimensional vector with the same size as the input traffic flow matrix. Both of these vectors together comprise the model input.

## 2. Methodology

### 2.1. Traffic Flow Matrix Generation

Many studies have employed sensor data (e.g., from loop sensors, cameras, and taxicab GPSs) to extract the average speed and traffic flow volume of a road segment and integrated those data with other information to estimate the future traffic status [20,21,22,23]. However, the sensors’ data coverage is limited by the cost of placing these sensors. Here, we adopt taxicab GPS trajectory data to construct the model input. This type of data source provides as much coverage as the number of probe vehicles allows and can provide a realistic sampling of an urban road network’s distribution. Moreover, the cost of obtaining these data is greatly reduced.

**Figure 1:**
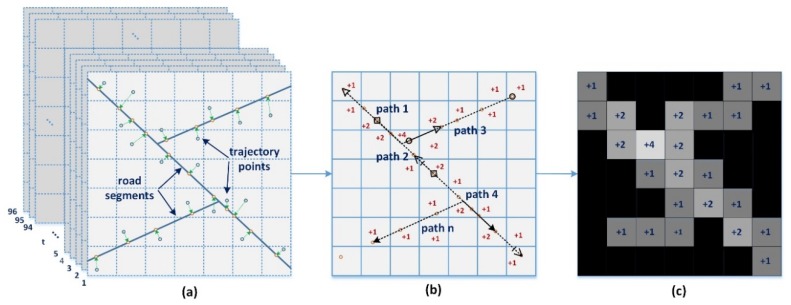
Data pre-processing procedure. (**a**) GPS trajectory slicing; (**b**) matching trajectory maps; (**c**) spatial intersection operation.

Traditional GPS trajectory processing methods focus on a vector road segment and take the road segment as a single processing unit to calculate its average speed or traffic flow volume. Unlike these routines, we process and output raster format traffic flow matrices or images. Each pixel value in the image represents the traffic flow volume of the road segment with smaller granularity, which retains more useful information from the source data. For example, each small component of a road segment actually has different traffic flow volumes at all times. Thus, vehicles sequentially move onto the main road from other roads over a period of time, and the front end occupies the greatest proportion of traffic flow volume. Inspired by this phenomenon, we utilize a lattice to split the source data and calculate each pixel value—the traffic flow volume of each small component of a road segment. The traffic flow matrix can be generated as follows:

(1) We first split the GPS trajectory data of each day into 96 slices; then, all these slices form a collection of cubic spatiotemporal trajectories, as shown in Figure 1a.
(2) We then match all these GPS trajectory points to the most suitable locations using the Hidden Markov Model (HMM) technique introduced in [24], as shown in Figure 1b.
(3) For each slice of the cube, we concatenate the sampling points of each taxi into a complete geometry termed a “path” that acts as a processing unit in the following procedure.
(4) We connect all these paths into a specific spatial resolution grid. Each grid unit represents the traffic flow volume of a small area during a 15 min time interval, as shown in Figure 1c.

Let T be a group of trajectories at the $i^{th}$ time interval. For a grid unit $C_{\left(i,j\right)}$ located at the $i^{th}$ row and the $j^{th}$ column, the traffic flow volume at time interval t is defined as

(1) $$X_{t}^{ij}=\sum_{T_{r}}\left|\left\{k>1|p_{k-1}\notin C_{\left(i,j\right)}\wedge p_{k}\in C_{\left(i,j\right)}\right\}\right|,$$

where $T_{r}:p_{1}\to p_{2}\to \cdots \to p_{\left|T_{r}\right|}$ is a trajectory in T, and $p_{k}$ is the geographical coordinates (e.g., the projected coordinates);$p_{k}\in C_{\left(i,j\right)}$ means that point $p_{k}$ lies within grid unit $C_{\left(i,j\right)}$ and vice versa; |⋅| denotes the cardinality of a finite set.

**Figure 2:**
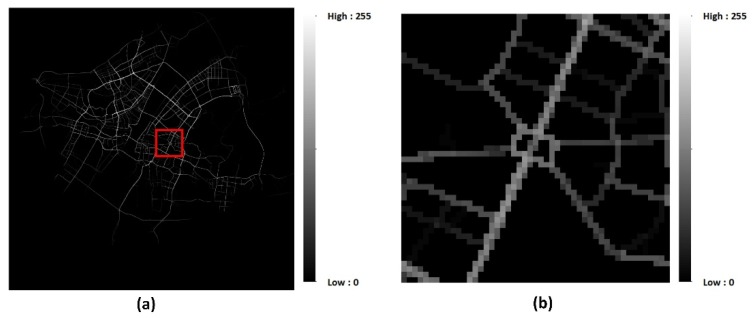
Sample traffic flow matrix. (**a**) Traffic flow volume at 12:00, 5 January 2015; (**b**) detailed view of the traffic flow matrix of (**a**).

At the $i^{th}$ time interval, traffic flow volume in all I×J regions can be denoted as a tensor $X_{t}\in {\mathbb{R}}^{I\times J}$. A sample traffic flow matrix of the research area is shown in Figure 2.

### 2.2. Model Structure of TFFNet

The Deep Convolutional Neural Network (DCNN) has been proven to be a state-of-the-art technique in many computer vision tasks, such as image recognition, object detection, image segmentation, etc. In this paper, we construct a DCNN model based on the Residual Network architecture, which can effectively model the spatiotemporal dependencies for traffic status evolution and incorporate external factors (e.g., weather, accidents, holidays) into the model.

Convolutional layers, pooling layers, and fully connected layers comprise the conventional convolutional neural network (CNN) model. The convolutional layer utilizes a large number of convolutional kernels to extract feature maps from the previous layer. The pooling layer is introduced to diminish the spatial dimensions of the current feature map by using average or max-pooling computation. The fully connected layers are the final part of a CNN model. Usually, the feature map and the fully connected layer are further filtered via an activation function [24,25,26]. Activation functions are helpful for improving a model’s non-linear fitting ability and effectively speeding up the convergence of the training process and greatly enhancing the model’s generalization ability.

Due to the existence of the vanishing-gradient problem, the network parameters cannot be tuned properly by the optimization algorithm, so deep models cannot achieve better performance than shallow ones [27]. Traditional CNN models only stack a few convolutional layers due to a lack of sufficient computing power. Deep residual learning allows CNNs to have a very deep structure of over 100 layers (with as many as 1000 layers) [28]. This method has been used in many modern DCNN frameworks and has produced state-of-the-art results for many challenging computer vision tasks. Formally, a residual unit with an identity mapping function can be defined as follows:

(2) $$X^{\left(l+1\right)}=X^{\left(l\right)}+ℱ\left(X^{\left(l\right)}\right),$$

where $X^{\left(l\right)}$ is input of the $l^{th}$ residual unit; $X^{\left(l+1\right)}$ is the output of the same residual unit; and ℱ is a learnable residual function, such as the bundle of 3 × 3 convolutional layers in [28]. The goal of residual learning is to learn an additive residual function ℱ with respect to $X^{\left(l\right)}$.

**Figure 3:**
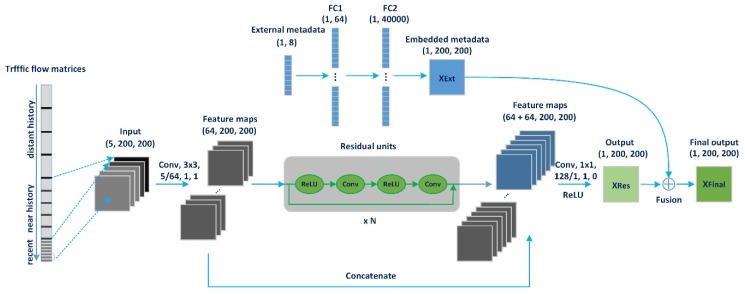
TFFNet architecture. Conv: convolution layer; FC: fully-connected layer.

Figure 3 presents the architecture of the traffic flow forecasting network (TFFNet), which is comprised of two components modelling spatiotemporal dependencies and external influences, respectively. As illustrated in the bottom part of Figure 3, we first transform the traffic flow volume for the whole city at each time interval into a 1-channel image-like matrix using the approach introduced in Section 2.1. Then, we organize those matrices along the time axis and divide the time axis into three fragments, denoting recent time, near history, and distant history. The traffic flow matrices representing the traffic status of the intervals in each time fragment are then extracted and concatenated and fed into the TFFNet to automatically learn hierarchical spatiotemporal dependencies. For example, to predict $X_{n}$, we extract the matrices from historical observations $\left\{X_{t}|t=0,\cdots ,n-1\right\}$, including the above three time fragments, to model temporal properties, including temporal closeness, period, and trend, and then concatenate those matrices to construct a new high-dimensional tensor $X_{in}\in \left(l_{c}+l_{p}+l_{q}\right)\times I\times J$, where $l_{c}$, $l_{p}$ and $l_{q}$ represent the length of the temporally dependent sequence. We feed the input data into the first convolutional layer to extract the intensive shallow and local features hidden in a temporally dependent sequence using a small-size convolution kernel. Next, we stack a series of residual units to extract deeper and multi-scale spatiotemporal features. Finally, we stack another convolutional layer to extract deeper and more global features and denote the model output as tensor $X_{Res}$. We then summarize the above three procedures as embedding, extraction, and prediction. These procedures constitute a complete prediction process. Inspired by ResNet [27,28], we add a shorter connection to the framework and concatenate the output feature maps from the first convolutional layer and the residual units together. This architecture alleviates the vanishing-gradient problem, strengthens feature propagation, and substantially supplements the local spatial structure information lost in stepwise forward computation. As illustrated in the top part of Figure 3, we adopt a two-layer fully-connected neural network to embed external factors into the hidden layer. The model feeds the one-hot alike feature vector and outputs a structured encoded tensor $X_{Ext}$ with the same dimensions as $X_{Res}$. $X_{Res}$ is further fused with $X_{Ext}$, and the final output is $X_{Final}$.

The main component (the bottom part of Figure 3) of TFFNet is composed of two sub-components: the convolution and residual unit. More details are described in the following section.

Convolution. A city’s road network usually consists of road segments of a very large size with inherent topological relationships. The traffic flow volume in each road segment may be affected by nearby and distant traffic status, which can be effectively handled by CNNs, which have shown their powerful ability to hierarchically capture spatial structure information [29]. The spatial dependencies of nearby road segments can be captured by applying shallow convolutional operations, whereas the distant ones must adopt a very deep architecture to capture global correlations. Thus, we build a model with many layers based on ResNet, which satisfies the actual demand for extracting the hierarchical spatial dependencies of road segments. Unlike a traditional CNN, TFFNet only uses convolutions and consumes more training time, but this makes for a much simpler architecture than traditional CNNs.

We extract the matrices from the recent time, near history, and distant history fragments of the historical observations to construct three temporal dependent sequences ($X_{c}$, $X_{p}$ and $X_{q}$, respectively). Then we concatenate $X_{c}$, $X_{p}$, $X_{q}$ into one high-dimensional tensor $X_{in}=\left[X_{c},X_{p},X_{q}\right]$. The mathematical formulations of $X_{c}$, $X_{p}$, $X_{q}$ can be denoted as follows:

(3) $$X_{c}=\left[X_{t-l_{c}},X_{t-\left(l_{c}-1\right)},\cdots ,X_{t-1}\right],$$

(4) $$X_{p}=\left[X_{t-l_{p}\cdot p},X_{t-\left(l_{p}-1\right)\cdot p},\cdots ,X_{t-p}\right],$$

(5) $$X_{q}=\left[X_{t-l_{q}\cdot q},X_{t-\left(l_{q}-1\right)\cdot q},\cdots ,X_{t-q}\right],$$

where $l_{c}$, $l_{p}$, and $l_{q}$ denote the lengths of the three temporally dependent sequences, and p and q represent two different types of periods. In our detailed implementation, p is set to one day to describe daily periodicity, and q is set to one-week revealing the weekly trend.

Spatial correlation can be calculated by moving the convolutional kernel in the traffic flow matrix, and a different band of input tensor $X_{in}$ can be merged by applying weighted summation. The first layer output of TFFNet can be represented as follows:

(6) $$X^{\left(1\right)}=f\left(\sum_{j=1}^{l_{c}+l_{p}+l_{q}}W_{j}^{\left(1\right)}*X_{j}+b^{\left(1\right)}\right),$$

where ∗ denotes the convolution operator, and f is a non-linear activation function (e.g., f(z):=max(0,z)) [24]; $W_{j}^{\left(1\right)}$, $b^{\left(1\right)}$ are the learnable parameters of the first layer, and $X_{j}\in X_{in},j=1,2,\ldots ,l_{c}+l_{p}+l_{q}$.

Analogously, the subsequent layer output of TFFnet can be deduced by applying similar operations. At the end of the TFFNet framework, the convolutional layer output is denoted as $X_{Res}$.

Residual unit. A DCNN model with a large number of layers will problematize the training process, though we can use the activation function and regularization techniques to ameliorate such problems [24,30,31]. However, we still require a very deep architecture to extract global spatial dependencies or city-wide spatial correlations. To overcome this problem, we introduce residual learning [27] in our model, which has been demonstrated to be very powerful for training a DCNN model with more than 1000 layers.

In our implementation, we mainly employ a residual unit that contains two combinations of ‘ReLU + Convolution (3×3 kernel)’. We also attempt to deploy batch normalization (BN) [30] before the activation function, ReLU. This combination has demonstrated its effectiveness among different ResNet derivatives [28]. Formally, we stack L residual units upon the first convolution layer—the final output $X^{\left(l+1\right)}$ can be denoted as follows:

(7) $$X^{\left(l+1\right)}=X^{\left(l\right)}+ℱ\left(X^{\left(l\right)};\theta^{\left(l\right)}\right),$$

where ℱ is the residual function, and $\theta^{\left(l\right)}$ are the parameters of the $l^{th}$ (l=1,2,…,L) residual unit.

External components. Traffic flows in the road network can be influenced by many complex external factors, such as weather and events [20]. The city’s transportation system behaves differently between weekdays and the weekend, especially during holidays. Severe weather also affects people’s travel behavior and can advance or postpone morning or evening rush-hour. Traffic accidents can lead to unexpected traffic jams, yielding changes in the surrounding road conditions. Let $E_{t}$ be the external factor feature vector at the predicted time interval t. In our implementation, we mainly consider holiday events and metadata (i.e., days of the week and weekday/weekend). As shown in Figure 3, we use two fully-connected layers to process the external factor feature vector $E_{t}$. The first layer is an embedding layer and is followed by an activation function. The second layer is a mapping layer aimed at converting the feature vector from a low-dimension to a high-dimension. Finally, the high-dimensional feature vector will be reshaped to the same size of the main component output $X_{Res}$. The output of the external component is denoted as $X_{Ext}$, whose learnable parameters are represented as $\theta_{Ext}$.

Fusion. Before outputting the final predicted value, we need to fuse the output of the above two components together. We directly add the output of the above two parts using matrix addition, as shown in Figure 3. The predicted value $\hat{X_{t}}$ can be defined as

(8) $$\hat{X_{t}}=\tanh\left(X_{Res}+X_{Ext}\right),$$

where tanh(⋅) is the hyperbolic tangent activation function.

TFFNet must be trained to predict the traffic flow volume at the $t^{th}$ time interval (i.e., $X_{t}$) from temporally dependent sequences and the external factor feature vector by minimizing the loss function. In our implementation, we use the mean squared error (MSE) as the optimization target:

(9) $$ℒ\left(\theta \right)=\|X_{t}-{\hat{X_{t}}\|}_{2}^{2},$$

where θ represents all parameters learned during the training process.

### 2.3. Training Process of TFFNet

Algorithm 1 summarizes the training process of TFFNet. We first construct training instances from the original dataset (e.g., traffic flow matrices and external factors). Then, we train the model via gradient-descent based back-propagation and an Adam stochastic optimization algorithm [32]. Given the temporal dependent sequences $X_{c}$, $X_{p}$, $X_{q}$, the external factor feature vector $E_{t}$, and the target value $X_{t}$ for any time interval t (1≤t≤n−1), we construct a training instance $\left(\left\{\left|X_{c},X_{p},X_{q}\right|,E_{t}\right\},X_{t}\right)$ and put it into a finite set S. During TFFNet’s training process, we initialize each model parameter θ using a uniform distribution with default values. Afterwards, we randomly select a batch of training instances $S_{b}$ from finite set S, and repeatedly feed them into the neural network training process. This optimization algorithm tries to find an optimal set of parameters θ by minimizing the objective function ℒ(θ), until the predefined stopping criteria is satisfied. After finishing the above iterative computation, we obtain the useable prediction model, ℳ.
**Algorithm 1** Training process for TFFNet**Input:** traffic flow matrices:    $\left\{X_{t}|t=0,\cdots ,n-1\right\}$;    external factors: $\left\{E_{t}|t=0,\cdots ,n-1\right\}$;    lengths of temporally dependent sequences: $l_{c}$, $l_{p}$, and $l_{q}$;    period parameter: p; trend parameter: q.**Output:** TFFNet model ℳ.1 2 3 4 5 6 7 8 9 10 11 12 13 14 S←∅ for all available time intervals t (1≤t≤n−1) do   $X_{c}=\left[X_{t-l_{c}},X_{t-\left(l_{c}-1\right)},\cdots ,X_{t-1}\right]$   $X_{p}=\left[X_{t-l_{p}\cdot p},X_{t-\left(l_{p}-1\right)\cdot p},\cdots ,X_{t-p}\right]$   $X_{q}=\left[X_{t-l_{q}\cdot q},X_{t-\left(l_{q}-1\right)\cdot q},\cdots ,X_{t-q}\right]$   // $X_{t}$ is the target value at time interval t   put a training instance $\left(\left\{\left|X_{c},X_{p},X_{q}\right|,E_{t}\right\},X_{t}\right)$ into S initialize model parameters θ **repeat**   randomly select a batch of training instances $S_{b}$ from S   find θ by minimizing the objective ℒ(θ) with $S_{b}$ **until** the stopping criteria is satisfied output the learned TFFNet model ℳ

## 3. Empirical Study

### 3.1. Experiment Settings

**Figure 4:**
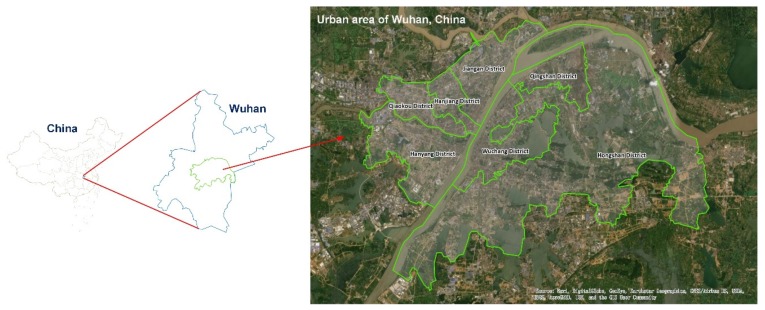
Location of the urban area in Wuhan, China.

**Datasets.** A private dataset for Wuhan, China, was used to evaluate our proposed model (Figure 4). This dataset contains trajectory, weather, and holidays information originating from government departments. Trajectories were provided by the transportation bureau, which contains information on 7000 vehicles’ trajectories from 1 April to 30 June 2017. The weather conditions and holidays information were acquired from open-access official websites (e.g., www.weather.com.cn and www.gov.cn). The dataset was separated into two parts: trajectories and external factors. More details can be found in Table 1.

**Figure 5:**
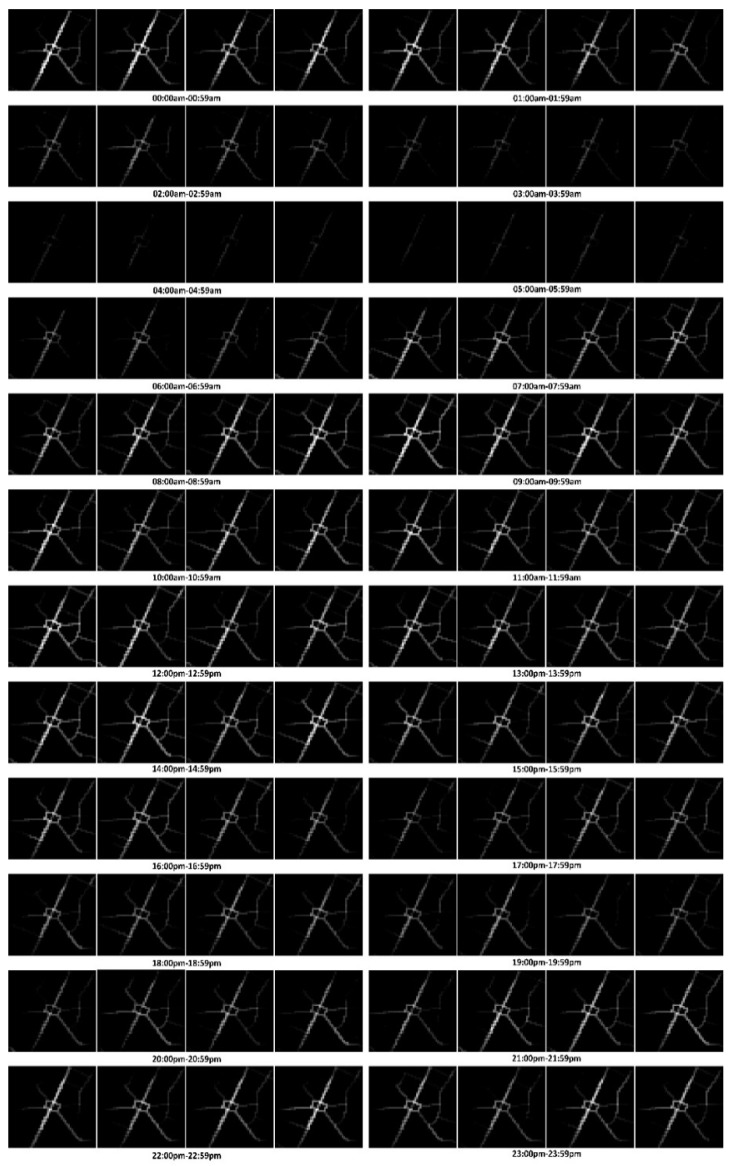
Traffic flow matrices of Hongshan Square on 1 May 2017.

**Preprocessing.** Trajectories were pre-processed and converted into traffic flow matrices according to the method mentioned in Section 2.1 before further processing. We acquired 8640 slices of the traffic flow matrix altogether. All of these data were used to construct training instances or training samples. A group of traffic flow matrices for the local region are illustrated in Figure 5.

We continue to construct the training instances from the traffic flow matrices and external factors according to the algorithm introduced in Section 2.3. To predict the traffic flow volume at t, a training instance $\left(\left\{\left|X_{c},X_{p},X_{q}\right|,E_{t}\right\},X_{t}\right)$ can be assembled as follows:

(1) For the temporal closeness sequence $X_{c}$, we extract the last three slices before the predicted time interval, i.e., $l_{c}=3$ and $X_{c}=\left[X_{t-3},X_{t-2},X_{t-1}\right]$.
(2) For period sequence $X_{p}$, we only utilize one slice of the traffic flow matrices, the same time interval as the previous day, i.e., $l_{p}=1$ and $X_{p}=\left[X_{t-96}\right]$.
(3) Trend sequence $X_{q}$ has a similar structure to $X_{p}$, and the same time interval for the same day as the previous week is used, i.e., $l_{q}=1$, and $X_{q}=\left[X_{t-672}\right]$.
(4) Then, we transform the external factors into a 1-dimensional feature vector $E_{t}$ using one-hot encoding, e.g., $E_{t}=\left[1,0,0,0,0,0,0,1\right]$ for 1 May 2015.
(5) $X_{c}$, $X_{p}$, $X_{q}$, $E_{t}$, and the target value $X_{t}$ constitute a training instance or training sample, which will be placed into a finite set S and fed into the neural network training.

All traffic flow matrices will be pre-processed following the above steps. Altogether, we achieved 7968 training instances derived from the original dataset. Afterwards, these instances can be directly fed into TFFNet for further training procedures. More details can be found in Table 2.

**Hyperparameters.** The deep learning library PyTorch (a popular deep learning programming framework) is used to build and train TFFNet. The learnable parameters of TFFNet are initialized using a uniform distribution with default parameters in PyTorch. TFFNet mainly uses 64 filters of 3×3 and 128 filters of 1×1. Table 3 provides the detailed structure of TFFNet, with four residual units. The Adam stochastic optimization algorithm is used to automatically adjust the model parameters during the neural network training procedure. In order to save GPU video memory, the batch size is set to a smaller value of 8. There are several extra hyperparameters in TFFNet, of which p and q are empirically set to one-day and one-week, respectively [21]. We set the lengths of the three temporally dependent sequences as $l_{c}=3$, $l_{p}=1$, and $l_{q}=1$. We select 80% of the training data for neural network training, and the rest (20%) are used for validating the model, which is applied to the early-stop training algorithm based on the best validation score. Subsequently, we continued to train TFFNet with the full training data for a fixed number of epochs (e.g., 100 epochs).

**Test Environment.** The experiments are mainly run on a workstation with a Tesla M60 GPU, Xeon E3 CPU, and 32 GB RAM. The deep learning library PyTorch 0.4 is used to build and train TFFNet. NVIDIA CUDA and cuDNN are both version 8.0.

**Evaluation Metric.** We measure our method by the Root Mean Square Error (RMSE) as

(10) $$RMSE=\sqrt{\frac{1}{z}\sum_{i}\left(x_{i}-{\hat{x}}_{i}\right)}$$

where $\hat{x_{i}}$ and $x_{i}$ are the predicted value and the ground truth, respectively, and z is the number of all predicted values.

### 3.2. Evaluation of Prediction Accuracy

In order to evaluate the effectiveness of TFFNet, we conducted some experiments to compare our method with four other typical methods. The historical average value (HA) is the simplest way to predict traffic flow. For example, the traffic flow for 12:45–13:00 on Monday could be predicted by computing all historical time intervals from 12:45 to 13:00 on Monday. As a typical time series problem, traffic flow can be predicted by the autoregressive integrated moving average (ARIMA), which is an effective tool for predicting future values. SAE is a neural network comprising multiple layers of autoencoders, where model inputs are encoded into dense or sparse representations before being fed into the next layer [19]. LSTM is an extension of recurrent neural networks (RNN) and has become popular because the architecture can deal with long-term memory and avoid the vanishing-gradient problem that traditional RNNs suffer from [33].

Table 4 shows the experimental results of the above four comparative methods versus TFFNet when applied to the testing dataset in a one-step prediction task. The results show that, in almost all circumstances, our proposed model outperforms the others in the testing dataset, suggesting that TFFNet can more effectively learn spatiotemporal dependencies from training instances, with a strong non-linear fitting ability for traffic prediction problems.

Both HA and ARIMA focus on each road segment of the whole road network. Hence, to predict network-wide traffic flows, a large number of independent models have to be built. In contrast, SAE and LSTM can yield network-wide traffic flows in one model with one or multi-step outputs. Regarding the ability to learn spatial dependencies, the above four methods all treat road segments independently and cannot effectively learn the topological relationships within the road network. This may be one possible reason why HA, ARIMA, SAE, and LSTM’s performance is inferior to that of TFFNet for the tested dataset. These models neglect the spatial correlations of traffic flows in different road segments (e.g., a traffic accident that occurs in one road segment will affect adjacent segments over a long period of time).

### 3.3. Results of Different TFFNet Variations

#### 3.3.1. Impact of Model Depth

To further explore the effectiveness of different depths of TFFNet, we test eight variations of TFFNet with different depths, and the experimental results are shown in Table 5. For example, TFFNet_16 has 16 residual units and fuses with external factors. To avoid GPU video memory overflow, we only test TFFNet_4 to TFFNet_34 in this paper.

We observe that all of these models are superior to the previous comparative methods. This further proves the effectiveness of the spatiotemporal dependency modelling approach introduced by TFFNet. Compared with the best comparative methods, TFFNet_16 reduces the RMSE to 14.07, which significantly improves prediction accuracy. Although TFFNet_20 achieves even better results than TFFNet_16, it consumes much more training time after stacking four more residual units. Significantly, TFFNet_24, TFFNet_30, and TFFNet_34 do not surpass TFFNet_16 during the test, which demonstrates that the deeper architecture still leads to a network degradation phenomenon and affects the model’s prediction performance. A much deeper architecture will consume more training time but will only gain limited improvement in prediction accuracy. Therefore, we consider the depth of the model in practice and make a trade-off between depth and performance.

#### 3.3.2. Impact of Fusion Policy

To check the effects of external fusion on the prediction results, we slightly adjust the structure of TFFNet and retrain the model. Table 6 shows the experimental results of the different fusion policies of TFFNet. TFFNet_16 achieves a higher prediction accuracy than TFFNet_16_noFusion, which demonstrates that external fusion, to some extent, improves model performance. For training time, TFFNet_16 requires only 6.3 more minutes than TFFNet_16_noFusion but can achieve a higher prediction accuracy. The city’s transportation system behaves differently between weekdays and the weekend, especially during holidays. Severe weather also affects people’s travel behaviour and can advance or postpone morning or evening rush-hour. Traffic accidents can lead to unexpected traffic jams, yielding changes in the surrounding road conditions. Thus, these factors are worth considering.

#### 3.3.3. Impact of the Input Structure

To evaluate the effects of temporal properties on the prediction results, we fine-tune the input data structure and feed the results to TFFNet_16 to train another two variations, TFFNet_16_CP and TFFNet_16_C. Compared with the architecture of TFFNet_16, TFFNet_16_CP does not use trend data, and TFFNet_16_C only consumes temporal closeness data. The experimental results show that TFFNet_16 performs best on the tested dataset. TFFNet_16_CP and TFFNet_16_C are inferior because they lack the necessary temporal properties. This further demonstrates that the temporally dependent modelling of traffic flow can significantly improve prediction accuracy, especially for coarse-grained time interval input data. Similar conclusions can be found in studies of the same field (e.g., [20,21], etc.). The experimental results are shown in Table 7.

We also carried out another experiment to test and verify the model’s generalization ability when applied to other research areas. Before predicting the future status of a traffic system, we constructed a few new training instances using the method introduced in Section 3.1 and continued to train the output prediction model ℳ for several epochs by utilizing the transfer learning policy, which greatly improves the performance of learning by avoiding many expensive data-labelling efforts [34]. Then, we output the final prediction model. We acquired a similar prediction result in the final experiment. Thus, we can conclude that TFFNet has a strong generalization ability for similar problems because the deep neural network has a strong non-linear fitting ability.

Based on the above discussion, useful conclusions can be drawn, as follows:

TFFNet outperforms other typical methods for testing datasets, which implies that it is useful for learning the spatiotemporal dependencies hidden in traffic flow matrices.

The fusion of external factors remarkably reduced RMSE during the evaluation, which implies that external factors could be properly fused with the prediction result to improve prediction accuracy.

A deep residual network provides the ability to build a CNN model with over 100 layers, which provides a good foundation for constructing a model used to extract hierarchical spatial features.

By adopting a transfer learning policy, TFFNet could be transferred and used in other research areas and shows a strong generalization ability for the traffic flow prediction problem.

## 4. Conclusions and Future Work

This paper proposes a deep-learning-based traffic flow prediction method that can model spatio-temporal dependencies by applying a fully convolutional architecture. With deep residual learning introduced into TFFNet, this method can utilize deep convolutional structures to extract hierarchical spatial features ranging from shallow to deep, thus allowing it to model spatial dependencies from near to distant regions. By extracting historical traffic flow matrices from recent times, near history, and distant history time segments, we can construct temporally dependent sequences and model temporal closeness, periods, and trends via multi-channel convolution computations. The fusion of external factors, to some extent, improves traffic flow prediction accuracy, especially for holiday events, and various metadata (i.e., for days of the week and weekday/weekend) are involved. We evaluate TFFNet and other baselines on a private dataset and further explore the impacts induced by different model depths, fusion policies, and input structures. The experimental results show that TFFNet and its different variations outperform other typical prediction methods on testing the dataset in a city-wide one-step traffic flow prediction problem, which is especially suitable for image-based format inputs and outputs.

This model possesses the following advantages: (1) end-to-end training reduces the dependencies of the existing model and its pre-experience and can yield a complex structured output; (2) the prediction accuracy can be fine-tuned by increasing or reducing the residual units when considering different application scenarios; (3) a multi-branch network architecture or ensemble learning policy makes the fusion of external factors feasible and effective.

However, there are some drawbacks to this method, especially regarding the difficulty in the model’s interpretability. Many studies concerning model interpretability have been carried out. Current works focus on the visualization of feature maps in hidden layers, showing that DCNN can extract hierarchical image features, from abstract to concrete and generalized to specialized features [35,36,37].

Real-time prediction is another issue worth discussing. ITS plays an important role in modern cities and places higher demands on the real-time prediction ability of the available prediction methods. TFFNet takes traffic flow matrices and external factors as its model input and outputs the future short-term traffic flow volume of the whole road network in a 15 min time span. When we finish the training process and output the pre-trained model, the factors affecting the real-time prediction ability are the traffic flow matrix generation and the encoding of external factors, which converts trajectories and external factors into model inputs. Before carrying out further predictions, we have 15 min to process the original data by employing a powerful distributed parallel computing platform, such as Hadoop, Spark, or Flink. In this way, TFFNet will be deployed in a cloud environment, computation resources will be adequately supplied, and the real-time prediction ability of the model will be guaranteed.

In the future, we will consider a more complicated model architecture, especially for modelling temporal closeness, periods, and trends to capture temporal dependencies more precisely. In addition, we will also consider how to handle sparse spatial traffic flow matrix inputs to reduce training time consumption and preserve topological relationships. Other data sources (e.g., mobile phone location data and bus credit card data) will be involved, and an appropriate fusion mechanism will be considered.

## Figures and Tables

**Table 1:** Original dataset details (holidays include adjacent weekends).

Dataset	Details	
Wuhan dataset	Time span	1 April to 30 June 2017
	Trajectories	From a government-sponsored program
	External factors	From open-access official websites
Part I: Trajectories	Sampling rate	60 s
	Floating cars	7 thousand
	Floating car types	Taxi, Bus, and Private car
	Trajectories	14 million
	Trajectory points	14 billion
Part II: External Factors	Weather conditions	16 types (e.g., Sunny, Rainy)
	Weekdays	65 days
	Weekends	26 days
	Holidays	9 days

**Table 2:** Training dataset details.

Dataset	Details	
Wuhan training dataset	Time span	90 days
	Time interval	15 min
	Traffic flow matrices	8640 (90 days × 96/day)
	Training instances	7968 (8640 − 7 × 96/day)
	Traffic flow matrix size	(200, 200)
	External factor feature vector size	(1, 8)

**Table 3:** Detailed structure of TFFNet with 4 residual units.

Layer Name	Input Size	Output Size	Filter Size
Conv 1	200×200, 5	200×200, 64	3×3, 64
ResUnit 1	200×200, 64	200×200, 64	$\left[\begin{array}{c} 3\times 3,\;64\\ 3\times 3,\;64 \end{array}\right]\times 1$
ResUnit 2	200×200, 64	200×200, 64	$\left[\begin{array}{c} 3\times 3,\;64\\ 3\times 3,\;64 \end{array}\right]\times 1$
ResUnit 3	200×200, 64	200×200, 64	$\left[\begin{array}{c} 3\times 3,\;64\\ 3\times 3,\;64 \end{array}\right]\times 1$
ResUnit 4	200×200, 64	200×200, 64	$\left[\begin{array}{c} 3\times 3,\;64\\ 3\times 3,\;64 \end{array}\right]\times 1$
Conv 2	200×200, 128	200×200, 1	1×1, 128

**Table 4:** Comparison of the different methods in the testing dataset.

Models	RMSE
HA	48.77
ARIMA	24.88
SAE	32.66
LSTM	20.38
TFFNet	18.34

Note: There are 4 residual units in standard TFFNet. The external fusion component is used. The model input incorporates 3 dependent sequences: temporal closeness, period, and trend.

**Table 5:** Comparison of the different model depths of TFFNet.

Models	Description	RMSE	Training Time (Minutes)
TFFNet_4	4 Residual Units	18.34	67.5
TFFNet_8	8 Residual Units	17.69	88.0
TFFNet_12	12 Residual Units	14.20	109.8
TFFNet_16	16 Residual Units	14.07	135.0
TFFNet_20	20 Residual Units	14.06	166.5
TFFNet_24	24 Residual Units	14.12	201.3
TFFNet_30	30 Residual Units	14.56	238.9
TFFNet_34	34 Residual Units	15.09	279.5

Note: TFFNet_* represent how many residual units are stacked in constructing the TFFNet model.

**Table 6:** Comparison of the different fusion policies of TFFNet.

Models	Description	RMSE	Training Time (Minutes)
TFFNet_16	With external fusion	14.07	135.0
TFFNet_16_noFusion	Without external fusion	15.77	128.7

**Table 7:** Comparison of the different input structure of TFFNet.

Models	Input Data Structure			RMSE	Training Time (Minutes)
	Temporal Closeness	Period	Trend		
TFFNet_16	✓	✓	✓	14.07	135.0
TFFNet_16_CP	✓	✓	✕	18.29	132.7
TFFNet_16_C	✓	✕	✕	19.64	130.2
